# Two sides of the same coin – an interview study of Swedish obstetricians’ experiences using ultrasound in pregnancy management

**DOI:** 10.1186/s12884-015-0743-5

**Published:** 2015-11-20

**Authors:** Annika Åhman, Margareta Persson, Kristina Edvardsson, Ann Lalos, Sophie Graner, Rhonda Small, Ingrid Mogren

**Affiliations:** Department of Clinical Sciences, Obstetrics and Gynecology, Umeå University, Umeå, Sweden; Department of Nursing, Umeå University, Umeå, Sweden; Judith Lumley Centre, La Trobe University, Melbourne, Vic 3000 Australia; Department of Women’s and Children’s Health, Karolinska Institutet, Solna, Sweden; Department of Medicine, Centre for Pharmacoepidemiology, Karolinska Institutet, Solna, Sweden

**Keywords:** Autonomy, Human rights, Obstetric ultrasound, Obstetrician, Pregnancy, Pregnant women, Prenatal diagnostics, Qualitative study, Sweden

## Abstract

**Background:**

The extended use of ultrasound that is seen in maternity care in most Western countries has not only affected obstetric care but also impacted on the conception of the fetus in relation to the pregnant woman. This situation has also raised concerns regarding the pregnant woman’s reproductive freedom. The purpose of this study was to explore Swedish obstetricians’ experiences and views on the role of obstetric ultrasound particularly in relation to clinical management of complicated pregnancy, and in relation to situations where the interests of maternal and fetal health conflict.

**Methods:**

A qualitative study design was applied, and data were collected in 2013 through interviews with 11 obstetricians recruited from five different obstetric clinics in Sweden. Data were analysed using qualitative content analysis.

**Results:**

The theme that emerged in the analysis ‘Two sides of the same coin’ depicts the view of obstetric ultrasound as a very important tool in obstetric care while it also was experienced as having given rise to new and challenging issues in the management of pregnancy. This theme was built on three categories: I. Ultrasound is essential and also demanding; II. A woman’s health interest is prioritised in theory, but not always in practice; and III. Ultrasound is rewarding but may also cause unwarranted anxiety.

**Conclusions:**

The widespread use of ultrasound in obstetric care has entailed new challenges for clinicians due to enhanced possibilities to diagnose and treat fetal conditions, which in turn might conflict with the health interests of the pregnant woman. There is a need for further ethical discussions regarding the obstetrician’s position in management of situations where maternal and fetal health interests conflict. The continuing advances in the potential of ultrasound to impact on pregnancy management will also increase the need for adequate and appropriate information and counselling. Together with other health care professionals, obstetricians therefore need to develop improved ways of enabling pregnant women and their partners to make informed decisions regarding pregnancy management.

## Background

Obstetric ultrasound is available in most parts of the world, but it is more extensively used in high-income countries than in low-income countries [1, 2]. An obstetric ultrasound examination can be done only once or at each pregnancy check-up, for medical reasons or for the expectant parents’ desire to ‘see’ their unborn child [3]. Ultrasound plays a significant role in assessment of gestational age, early detection of multiple pregnancy, localization of the placenta, detection of fetal malformation, estimate of fetal size and amniotic fluid amount [4]. It has also been shown that careful monitoring of fetal health by use of Doppler ultrasound can reduce obstetric intervention and decrease risk for intrauterine fetal death in high risk pregnancy [5]. Still, there is no evidence that routine scans reduce adverse outcomes for newborns in general [6].

The ultrasound scan is very attractive to pregnant women [3]. They expect the scan to confirm the wellbeing of the fetus, and to provide a visual encounter with their ‘baby’ [7, 8]. For expectant fathers the ultrasound can be a confirmation of new life [9]. Viewing the fetus through ultrasound is also said by expectant fathers to make the fetus more real to them [9, 10] and it assists them to adjust to the situation as a prospective parent [11]. While uncertain and unknown aspects of fetal health are recognised to create anxiety in women, this anxiety can lessen when women view the ultrasound image and receive information that “everything is normal” [12]. Additionally, it is shown that even non-medical ultrasound examinations may be considered by expectant parents as an assurance that the fetus is healthy [13]. The extended use of ultrasound in pregnancy surveillance has also had an impact on the conception of the fetus as a patient with its own health interests and needs [14]. This has raised concerns regarding women’s autonomy and reproductive freedom during pregnancy [14, 15] as methods for treatment of fetal conditions continue to develop [16].

In Sweden, fetal screening and diagnostic procedures are regulated in the Swedish Genetic Integrity Act (2006:351) where it is stipulated that prenatal screening and diagnostic examinations are voluntary [17]. Further, the law specifies that all pregnant women shall be offered general information about all routine fetal diagnostic examinations provided in antenatal care [17]. The national guidelines for antenatal care also state that the information provided shall enable women to make informed decisions concerning fetal screening and diagnostic procedures [18]. Still, it is acknowledge that the preparing information about obstetric ultrasound provided by antenatal health care midwives in Sweden can be deficient which might hinder women’s autonomy in decision making [19].

### Outline of the CROCUS study

This study is part of the CROss Country Ultrasound Study (CROCUS) which is an international project aiming at investigating midwives’ and obstetricians’ experiences and views of the use of ultrasound, and maternal and fetal roles and rights. The CROCUS study is being undertaken in a number of high-income and low-income countries in Europe, Africa, Asia and Oceania. In this paper, Swedish obstetricians’ experiences and views are investigated.

The specific purpose of this study was to explore Swedish obstetricians’ experiences and views on the role of obstetric ultrasound particularly in relation to clinical management of complicated pregnancy, and in relation to situations where the interests of maternal and fetal health conflict.

## Methods

### Study design

A qualitative study design was applied, and data were collected through individual interviews. An inductive approach was used in the analysis of data [20].

#### Setting

Swedish public health care insurance covers the costs of antenatal care, which includes an offer of a second trimester fetal anomaly ultrasound scan free of charge, and 98 % of pregnant women in Sweden accept this offer. Women are usually accompanied by their partner during the examination [7] that is performed at 18 to 20 weeks of gestation. This ultrasound examination is most often performed by specially trained midwives who usually also conduct additional ultrasound examinations for assessment in later pregnancy such as estimation of fetal growth and amount of amniotic fluid. Women with known medical conditions that require extended fetal examinations and consultation are referred to an obstetrician for the routine ultrasound scan. Midwives conducting ultrasound examinations may also refer women to an obstetrician for a second opinion when they detect or suspect anything abnormal about the fetal condition or the intra-uterine environment. The Swedish Radiation Safety Authority prescribes that pregnancy ultrasound may be performed for medical reasons only [21]. However, outside the public health care system, ultrasound examination of the fetus is available in private clinics where women can undergo additional examinations at their own request and expense.

#### Participants

Participants were recruited from five purposively selected obstetric clinics located in different parts of Sweden. Diversity was sought regarding the size of clinics and level of health care. Variety in gender, age, professional qualifications and work experience of participants was also sought. Inclusion criteria for participation were being an obstetrician, performing obstetric ultrasound examinations on a regular basis, either as a major work task or as part of general obstetric care, or using the results of obstetric ultrasound in clinical management of pregnant women. Names and contact details of eligible obstetricians were obtained via the heads of the obstetric departments or through other health care professionals with extensive knowledge of the local clinic.

Eleven obstetricians meeting the inclusion criteria were included in this study. Initially, thirteen obstetricians were contacted via an e-mail that presented written information about the study and an invitation to participate. All 13 obstetricians who were initially approached agreed to participate, but two of the eligible participants withdrew later when no suitable time for the interview could be found. Written informed consent was obtained from each participant prior to the start of the interview.

The participants were between 33 and 63 years of age (mean age 48 years). Three were males and eight were females, and they reported between 2 and 30 years (mean 16 years) of experience in the field of obstetrics. Their level of training ranged from basic courses in fetal ultrasound examinations including doppler examinations to more advanced training in examination of the fetal heart, and fetal therapy. The clinics where the participants worked at the time of the interview, varied from general obstetric clinics to more specialised referral obstetric clinics, and the number of births at the clinics ranged from approximately 850 to 4800 births annually.

#### Data collection procedures

The interview guide was developed by the research team after a thorough review of the scientific literature [14, 22–28], and the interview guide was also based on the authors’ clinical experiences. Beyond the general scope, the literature review focused specifically on research on maternal and fetal role and rights in relation to the use of ultrasound in pregnancy management, and the development of fetal medicine. The interview guide was pilot tested in a previous study [29], and was used in the current study to ensure that the same set of topics were covered in all interviews, although not in any specific order. The informants were encouraged by the interviewer to speak freely regarding their experiences and views related to obstetric ultrasound. Probing questions were used throughout the interviews to gain a thorough description of participants’ experiences and views. However, any topic in the interview guide that was not spontaneously raised by the participants, was brought up by the interviewer. The key domains in the interview guide are presented in Table 1.

**Table 1:** Key domains in the interview guide

Key domains	
The obstetricians’ views/experiences of:	
•	The importance/value of obstetric ultrasound for clinical management of complicated pregnancy.
•	Clinical situations where the interests of maternal and fetal health have been in conflict.
•	Whether the woman may be considered to act as an instrument for fetal treatment.
•	The importance of obstetrical ultrasound in comparison to other surveillance methods during complicated pregnancy.
•	If/when the fetus can be regarded as a person.
•	Situations where the fetus has been regarded a patient with his/her own interests.
•	Their professional role in relation to other occupational groups working with obstetric ultrasound examinations or the outcomes of these examinations.

The interviews took place from October to December 2013 and were conducted in a place chosen by the interviewees, in most cases their own office. All participants completed a short questionnaire on background characteristics including questions regarding sex, age, professional qualifications and professional experience of obstetrics and obstetric ultrasound examinations. MP performed nine of the face-to-face interviews and IM performed two of the interviews.

The interviews were all digitally recorded and lasted between 33 and 48 min (mean time 41 min). After 11 interviews were performed the two interviewers examined the richness and diversity of the data obtained. They concluded that further interviews were unlikely to provide any new information.

#### Data analysis

Data were analysed using qualitative content analysis [30]. First, two members of the research team read all interviews to get a sense of the whole (AÅ and IM). The researchers then discussed their general impressions and emerging content areas. Data addressing the aims of this study were then coded by AÅ and selected parts were also coded by IM. AÅ and IM compared the codes for similarities and differences, grouped them into content areas and subsequently into preliminary categories and sub-categories. These codes, sub-categories and categories were then reviewed by AÅ, IM and MP and uncertainties in interpretation were discussed between the tree authors until consensus was reached. An overall theme, three related categories and seven sub-categories emerged in the analysis. The descriptions of the categories and sub-categories were then reviewed by the other co-authors KE, AL, SG and RS and some additional changes were made for clarity.

Ethical approval for this study was obtained from The Regional Ethics Committee in Umeå, Sweden, (Reference 2013/189-31).

## Results

### Two sides of the same coin

A main theme *‘Two sides of the same coin’* emerged from the overall analysis. On one hand, the participating obstetricians expressed great satisfaction with the benefits of obstetric ultrasound as a surveillance tool; on the other hand, they also raised concerns about some negative consequences of the use of ultrasound for the pregnant woman, the fetus and themselves as obstetricians. The theme was built on three categories: I. Ultrasound is essential, and also demanding; II. A woman’s health interest is prioritised in theory, but not always in practice; and III. Ultrasound is rewarding but may cause unwarranted anxiety. These categories are described in Table 2, together with their related sub-categories. Quotes from the interviews are presented to illustrate the results.

**Table 2:** Theme, categories and their subcategories

Theme	Category		Subcategory
Two sides of the same coin	I.	Ultrasound is essential and also demanding	A most valuable tool that is much relied on
			Raised expectations and demands on the *obstetricians’ operational* and counselling skills
			Women’s autonomy need to be guarded
	II:	A woman’s health interest is prioritised in theory, but not always in practice	The woman’s health should be our first priority
			The fetus becomes a person/patient via the ultrasound screen
			Pregnant women may suffer for the sake of the fetus
	III.	Ultrasound is rewarding but may cause unwarranted anxiety	Rewarding both to obstetricians as well as the expectant parents
			Ultrasound may also cause unwarranted anxiety

### I. Ultrasound is essential, and also demanding

This first category describes the obstetricians’ views on the value of obstetric ultrasound, its significance in obstetric care, and also their experiences regarding the increased demands that the extended use of ultrasound had brought. This included increased demands on the obstetricians’ operational skills and the need for advanced ultrasound training, as well as demands on their counselling skills.

#### A most valuable tool that is much relied on

The obstetricians considered ultrasound to be a very important tool for themselves in their work, but also for the expectant woman’s and her partner’s experiences in pregnancy. Ultrasound was regarded as especially important in the management of complicated pregnancy enabling assessment for example, of the optimal time of delivery.

*‘Mothers with severe preeclampsia in early pregnancy et cetera, and ultrasound is of course absolutely crucial then… how we manage the pregnancy, plan for the delivery’.* (Participant no 9)

Although ultrasound was considered essential to obstetric care, there were concerns raised among the obstetricians that they themselves, as well as the expectant parents, might be overconfident with regard to the outcome of the ultrasound examination. The high reliance on the results from ultrasound imaging was said to make expectant parents perceive ultrasound results with no evidence of deviations as an assurance that the fetus was healthy, although that might not be the case. Moreover, a strong focus on the results from ultrasound examinations was feared to decrease attention on other clinically important maternal parameters, such as surveillance of blood pressure and proteinuria.

*‘The assessment of preeclampsia of course involves my performing an ultrasound, and I do that. Then if the ultrasound looks fine, well that is good. Then I talk to the patient as if everything is fine and dandy, and then we look at her urine test and oops, so we do a check of her blood pressure and so…. I have to admit that I can fall into that trap.’* (Participant no 4)

#### Raised expectations and demands on obstetricians’ operational and counselling skills

The increased capacity of ultrasound technology and its extended use were recognised to have created increased demands on the operational ability of the professionals conducting the examination, and a need for more comprehensive training. There were concerns related to risk of missing deviations of significance and participants stressed that incorrect assessment could result in adverse fetal outcome. Further, the participants felt that there were high demands placed on their own counselling skills when informing expectant parents about abnormalities identified on ultrasound examination. Obstetricians at small clinics expressed special concern because they lacked senior colleagues to consult when ultrasound findings were of unclear significance and they felt unable to inform the women and their partners appropriately.

*‘It* [fetal assessment] *is more complicated, everything is more complex. (…) It places great demands on one’s knowledge, one’s ability to take care of the patient or to take care of and inform, and manage cases in the best possible way’.* (Participant no 8)

There were also concerns among the obstetricians about making incorrect medical assessments of the ultrasound or missing conditions essential for fetal health, especially when the woman was overweight. Moreover, it was felt that ultrasound images of poor quality led to disappointment for expectant parents when they were not provided the clear picture they had expected. Still, the obstetricians reported that expectant parents most commonly were very pleased with any opportunity to see their “baby” on the screen. Some of the obstetricians also admitted that at times when there was no medical indication for a fetal ultrasound examination, they did perform an ultrasound examination in order to calm an anxious couple.

*‘Sometimes you use it as ultrasound ‘treatment’* [for maternal anxiety]; *you don’t know what to do, so you put on the probe.’* (Participant no 1)

#### Women’s autonomy needs to be guarded

The obstetricians found it important to respect women’s right to make their own decisions; though they felt that their capacity to support women in making informed choices was sometimes limited by a woman’s lack of knowledge about the purpose and the potential of the examination. Protecting women’s autonomy was considered to be especially difficult when there were language barriers and no interpreter was available, as the obstetrician could not provide the information needed. It was also stressed that lack of knowledge could limit the possibility for women to make informed choices, not only about having a screening test or not, but also regarding possible additional fetal examinations or treatments. This was perceived as an ethical dilemma in the practice of ultrasound.

*‘When there is a just perceptible increased risk for some deviation and then you send the patient to a second examination without her asking for it. It’s quite a common problem, an ethical dilemma. For whom are we doing this and (…) are you prepared to face the consequences?’* (Participant no 9)

Some claimed that all information obtained from the ultrasound examination should be revealed to the expectant parents.

*‘I believe that if you find something then you have to inform the patient about it. Isn’t that actually fundamental (…) it’s the patient’s body, the patient’s fetus.’* (Participant no 7)

Moreover, occasional situations where expectant women requested early termination of pregnancy because of a minor fetal aberration, for example a cleft lip and palate, were considered ethically challenging to deal with, although it was agreed that a woman has the right to choose such an action.

### II. A woman’s health interest is prioritised in theory, but not always in practice

This second category depicts the obstetricians’ perceptions about how fetal assessment through ultrasound could affect clinical management during pregnancy, as well as their experiences of women’s health status versus fetal health status in decision-making and clinical management based on the results of obstetric ultrasound. The category describes the contrast between the obstetricians’ theoretical perspective, i.e. that women’s health should be the first priority in situations where both fetal and maternal health are at stake, and situations when obstetricians in their clinical management let women risk their own health for the sake of their fetus.

#### The woman’s health should be our first priority

The participants all agreed that in situations where a woman’s physical condition required medical treatment or premature delivery, her wellbeing should be prioritised regardless of whether it could entail adverse fetal health outcome. It was argued though that caring for the fetus was an important part of the obstetricians’ care of the pregnant woman and thereby their responsibility also to safeguard the health of the fetus. Dealing with these situations was described as a balancing act that at times made obstetricians agree to postpone delivery for the sake of fetal health, although this measure might not be the best option for the pregnant woman herself. However, the obstetricians reported that most women urged them to do “everything possible” to promote the health of the expected child, even if it implied suffering or increased health risks for the woman herself.

It was acknowledged though that the obstetric ultrasound examination put considerable focus on fetal health. This focus on the fetal condition was also said to have led to an increased involvement of neonatologists and other paediatric specialists, not only in planning for postnatal care but also in discussions regarding the optimal time for delivery and mode of delivery.

*‘When there is something, for example a heart defect that you can help the baby with, when it is born and so. Then the focus will shift to optimise* [the outcome] *for the baby, both during pregnancy and delivery.’* (Participant no 8)

#### The fetus becomes a person/patient via the ultrasound screen

Even though the status of the fetus as a person was claimed to be related mainly to time of fetal viability, it was acknowledged that the ultrasound image created an early sense of the fetus as a person. Some declared that in certain situations they adapted their own view to the expectant parents’ view of the fetus as being a “baby”, also before the time of viability.

*‘When you see a head, arms, legs,* [fetal] *movements then it* [the fetus] *becomes much more of a person, and with today’s machines you can see it very early, at week eight sometimes’.* (Participant no 1)

The obstetricians did believe it was their responsibility to safeguard the health of the fetus and that the ultrasound examination was an important tool for achieving this. They also thought that women most often complied with health professionals’ recommendations regarding monitoring and medical treatment for the sake of the fetal health. In rare cases, pregnant women’s wishes were said to be at odds with the health professionals’ beliefs about what was best for the fetus and that these situations were experienced as very frustrating by the obstetricians.

*‘There was a baby that was growth restricted who had umbilical cord blood flow classification one to three et cetera, but she* [the expectant woman] *would have none of it …, or felt that she knew better what was best for the child, as well. So it was not that she didn’t care about the baby but that she felt she knew better. And that is a very difficult situation.’* (Participant no 2)

#### Pregnant women may suffer for the sake of the fetus

Women were said to be strongly motivated and gave their consent to almost any type of treatment of fetal conditions that the obstetricians suggested, although the medical measure might be extremely stressful or even painful for the women. The opposite situation, where women declined treatment for the sake of the fetus, was experienced only rarely, and some obstetricians had had no such experience.

*‘I have never met an expectant mother who has hesitated to expose herself to something that might be harmful to her health as long as it benefits the fetus,’* (Participant no 5)

More commonly the participants reported situations when they as professionals had to decide, sometimes against the woman’s will, to discontinue treatment when side effects threatened the pregnant woman’s own health.

*‘Many women are prepared almost to push themselves over the precipice for the sake of their child. So it’s necessary that I put an end to it, somehow, so it won’t go too far’.* (Participant no 8)

It was suggested however that some pregnant women might feel that it was their duty, as an expectant mother, to go through medical tests and treatment for the sake of their ‘baby’ although they did not really want to do so. This situation was not perceived as common. Some also thought that obstetricians themselves might minimize any related risk to the woman’s health when informing the expectant parents about clinical management of adverse fetal conditions. To treat the fetus, or to postpone delivery, when it entailed risks for the pregnant woman’s health, was considered unethical. Still, obstetricians recognised that pregnant women sometimes were exposed to risks in association with interventions aiming to improve the fetal condition. Some also questioned if risking the woman’s wellbeing was beneficial for the baby in the long run.

*‘You could say that sometimes you might want to focus on the child even when there are some morbidity risks for the woman,… and where it might be of, dubious benefit for the child. When we might not help the child that much. But it’s very rare that you meet a women who isn’t willing to take that risk.’* (Participant no 11)

### III. Ultrasound is rewarding but may cause unwarranted anxiety

This third category describes the contrasts between the great satisfaction that obstetricians commonly experienced in relation to their use of ultrasound during pregnancy and the frustration they felt in problematic situations where the results from the ultrasound examination caused unwarranted anxiety for expectant parents.

#### Rewarding both to obstetricians as well as the expectant parents

The obstetricians described pregnancy ultrasound as a gratifying and often also an enjoyable part of their work and they appreciated the tool for the new possibilities it heralded as the capacity of the ultrasound machines constantly developed.

*‘It is a fantastic opportunity to be able to peek in there* [in the uterus] *in a way. (…) you see, I like to go diving (…) and I can feel it’s a bit like going down there into the water, into the uterus and there is that little creature in there with its air tank tied to its mother’.* (Participant no 6)

Additionally, conducting pregnancy ultrasound examinations was said to create a positive contact between the obstetrician and the expectant woman. It was suggested that these positive experiences might make obstetricians do more ultrasound examinations than is justified clinically. Providing the expectant couple with an ultrasound image was said to be a source of much appreciation from the woman and her partner. On the other hand, when the expectation of a clear image was unfulfilled, the obstetricians could experience great disappointment from the woman and her partner. Opinions among participants differed however, on whether it was acceptable or not to perform an ultrasound examination only to satisfy expectant parents. Some considered it important to be restrictive on this point, while others said that they did not hesitate to perform an ultrasound just to confirm that the fetus “was well”, for their own sake, but also to reduce expectant parents’ anxiety about fetal wellbeing.

*‘You don’t know what to do and so you put on the probe and sometimes a few too many ultrasounds are done without any indication.’* (Participant no 11)

It was also admitted though that an ultrasound examination without medical indication might just create a false sense of security.

#### Ultrasound may also cause unwarranted anxiety

Some obstetricians pointed out that people could respond very differently when receiving information either about minor or major fetal aberrations. It was recognised too that ultrasound findings of uncertain significance for the health of the fetus could create much unwarranted anxiety in expectant parents and this made counselling regarding such findings complicated. In these circumstances some queried how helpful it is to inform expectant parents of every detail of the findings.

*‘We detect vague findings sometimes and it’s difficult to tell what significance they might have for the unborn child, and then you have given rise to a few concerns in the parents. You might have destroyed a whole pregnancy by this.’* (Participant no 8)

Counselling regarding unclear ultrasound findings was perceived to be particularly difficult when the expectant parents had not been given sufficient information beforehand regarding the potential of the ultrasound examination, or when it was performed without medical indication. A particularly challenging situation was said to occur when findings that the obstetrician did not consider as severe resulted in termination of the pregnancy at the woman’s request.

*‘The only thing that sets me off are patients where we find something that I feel is quite trivial and who want to terminate the pregnancy. (…) I can respect their decision but then you can feel that, what if I had not had to see this? So much the better it would have been. It was truly unnecessary. Clubfoot is a typical example’* (Participant no 10)

Moreover, the obstetricians realised that expectant parents were very attentive to health professional reactions during the ultrasound examination, and felt that the slightest ambiguity revealed by the ultrasound operator could create anxiety in the expectant parents. The obstetricians were also aware that the emission of energy from the ultrasound machine can be potentially harmful for the fetus and examinations should therefore not be performed without medical indication. Still, because of the uncertainty of the evidence of harm, this was not taken much into account in their daily practice. Neither did it influence their ultrasound management in any major way.

*‘Then it is a bit like this with the effects* [thermal effect in fetuses] *of ultrasound, and so, also with the Doppler ultrasound and such; you don’t think about it much in everyday situations.’* (Participant no 2)

## Discussion

This study aimed to explore Swedish obstetricians’ experiences and views on the role of obstetric ultrasound with a focus on clinical management of complicated pregnancy and situations where the interests of maternal and fetal health conflict. We have also reported on a broader spectrum of issues related to ultrasound examination, given that participants themselves raised these issues during the interviews.

The main category ‘Two sides of the same coin’ illustrates the overall finding from this interview study, that the obstetricians viewed the ultrasound as an essential and much valued tool in obstetric care simultaneously as it had given rise to several challenging issues for obstetric practice. The challenges described were mainly related to the increased focus on fetal health as the fetus became a patient via the ultrasound image, which sometimes entailed dilemmas in decision-making and less focus on the health of women. This focus also raised expectations and demands on the obstetricians’ operational and counselling skills.

Our earlier study of Australian obstetricians’ experiences and views described the obstetric ultrasound as an invaluable tool for surveillance and management during pregnancy [29], a perspective consistent with these results from Sweden. The development of ultrasound equipment and its widespread use has however, also brought new challenges for professionals performing the examination. The most challenging issue described by participants in this current study concerned the balancing of ultrasound as a medical measure for the benefit of fetal health and the possible risks to pregnant women when acting on the findings.

### Dealing with conflicting health interests between the pregnant woman and the fetus

It is acknowledged that major ethical issues can occur when the health interest of the pregnant woman is in conflict with the health interest of the fetus [15]. Our findings showed a unanimous view among obstetricians that the woman’s health interests should be prioritised in case of conflicting maternal and fetal health interests. There were situations reported in our interviews where pregnant women had requested the obstetrician to “do anything possible” to enhance fetal outcome, although this might negatively affect the woman’s own wellbeing. Obstetricians found such situations most difficult to deal with. While respect for patient autonomy is one of the core tenets in health care, it is argued that this does not mean that health professionals have to accept demands from patients for inappropriate care [31]. Accordingly, the responsibility of the obstetricians is not to always to comply with the pregnant woman’s request. It has been claimed though that women’s right to refrain from treatment should be respected even when it might not be best for the fetus [26], which corresponds with the opinions expressed by the obstetricians in our study. Although rare, there were situations described where pregnant women declined treatment for the sake of the fetus and this could also be very challenging for obstetricians to handle.

Along with the development of fetal treatment and postnatal care there has been an increased involvement of paediatricians in consulting pregnant women concerning prenatal decisions [32]. A multidisciplinary approach to both prenatal diagnostics, pregnancy management and counselling pregnant women regarding management, has also been suggested to enhance pregnancy outcome [33]. Although pregnant women ask the obstetrician to “do anything possible” to enhance fetal outcome and are strongly motivated to participate in treatment to enhance the fetus condition as our results suggest, this does not relieve the obstetricians from their responsibility to safeguard the woman’s health and autonomy. However, there may be divergent ethical attitudes between obstetricians and paediatricians towards termination of pregnancy, and a discrepancy in attitudes between these two professional groups regarding pregnant women’s obligations towards the fetus [34]. Such differences in attitudes might influence the clinical counselling of women regarding management of pregnancy when fetal complications are detected [35]. This in turn has also been suggested to affect pregnant women’s autonomy [34].

#### A need to develop counselling

In contrast to the obstetricians’ great appreciation of ultrasound, our results also show a strong concern among the obstetricians that expectant parents lacked preparedness for the potential results from the ultrasound examination and that this sometimes caused unwarranted anxiety. Information about obstetric ultrasound is provided by the health care system, where midwives in antenatal care are the main providers of information and support during pregnancy [18]. If extended counselling or examinations are needed, expecting couples are referred to an obstetrician [18]. Still it seems that this information does not prepare expectant parents for the possibility of identifying minor aberrations or producing uncertain findings [36, 37]. A lack of understanding among expectant women regarding the potential of ultrasound examination has also been reported previously [38, 39]. Moreover, the counselling regarding prenatal ultrasound screening offered by antenatal health care professionals in Sweden has been suggested to be deficient [19, 40] which may impact negatively on women’s autonomy in decision making regarding prenatal screening and diagnostics.

The general offer of ultrasound screening in the second trimester has led to a situation where almost every pregnant woman (98 %) in Sweden accept this offer. Consequently this examination is no longer experienced as optional [36, 37], and many pregnant women already understand before their first visit to the midwife, that an ultrasound examination is done routinely in the second trimester [20]. Given the strong social expectations about having an obstetric ultrasound [8, 41], information on the medical potential of the examination might be considered less important. Still, the diversity of fetal conditions which currently can be evaluated through ultrasound increases the demands on both the ultrasound operators and other antenatal health care professionals as the information and the content in counselling become more complex [42].

As the ultrasound machine’s capacity develops and new options for prenatal tests become available the requirements on information and counselling in antenatal care will probably increase in countries where these options are available, therefore use of alternative methods of counselling might be needed. Patient decision aids have shown potential to enhance informed decision making both when used as a supporting tool during counselling and for patient use prior to a medical appointment [43]. It is recognized that there are challenges related to implementation, that targeted efforts may be needed to establish new routines for decision support in health care [44]. Furthermore, decisions to attend or not to attend prenatal screening are constructed in a social context were norms and expectations from society can determine women’s choices [45]. When informing expectant parents regarding pregnancy ultrasound and other prenatal tests such factors need to be taken into account.

#### Strengths and limitations

To strengthen credibility in this study we recruited participants from five different obstetric clinics. Additionally, the participants differed in characteristics such as age, gender and working experiences in obstetric practice. To promote transferability, we paid careful attention to describe both the typical and atypical views expressed by the obstetricians. Furthermore, having a clear decision trail through the analysis process enhanced dependability [30]. Nevertheless, our results are related to the Swedish setting and culture, and the organisation of Swedish obstetric care. It is likely though that many of the aspects described in our results are transferable to other high income Western societies.

## Conclusions

The widespread use of ultrasound in obstetric care has entailed new challenges for clinicians due to enhanced possibilities to diagnose and treat fetal conditions, which in turn might conflict with the health interests of the pregnant woman. There is a need for further ethical discussions regarding the obstetrician’s position in management of situations where maternal and fetal health interests conflict. The continuing advances in the potential of ultrasound to impact on pregnancy management will also increase the need for adequate and appropriate information and counselling. Together with other health care professionals, obstetricians therefore need to develop improved ways of enabling pregnant women and their partners to make informed decisions regarding pregnancy management.
